# The effect of magnesium on the reversal of rocuronium-induced neuromuscular block with sugammadex: an ex vivo laboratory study

**DOI:** 10.1186/s12871-019-0734-6

**Published:** 2019-05-01

**Authors:** Ákos I. Fábián, Vera Csernoch, Edömér Tassonyi, Marianna Fedor, Béla Fülesdi

**Affiliations:** Department of Anaesthesiology and Intensive Care, University of Debrecen, Faculty of Medicine, Nagyerdei krt. 98, Debrecen, 4012 Hungary

**Keywords:** Phrenic nerve-diaphragm preparation, Neuromuscular block, Rocuronium, Magnesium, Sugammadex

## Abstract

**Background:**

Magnesium dose-dependently potentiates the effect of non-depolarizing neuromuscular blocking agents. We investigated whether the potentiation of rocuronium-induced blockade by magnesium reduces the effect of sugammadex in an ex-vivo environment and how this influences the safety margin of reversal.

**Methods:**

Phrenic nerve – hemidiaphragm tissue preparations were isolated from male Wistar rats. The specimens were suspended in a tissue holder that allowed registering muscle contraction amplitude following electrical stimulation of the nerve. Concentration-response relationships were elucidated for magnesium, as well as for rocuronium and sugammadex.

**Results:**

The mean (95% confidence interval [CI]) half effective concentrations (EC_50_) of rocuronium in the presence of magnesium 1 mM or 1.5 mM were 7.50 μM (6.97–8.07 μM) and 4.25 μM (4.09–4.41 μM), respectively (*p* < 0.0001). Increasing magnesium from 1 mM to 1.5 mM during reversal of rocuronium-induced block increased the mean (95% CI) EC_50_ of sugammadex from 3.67 μM (3.43–3.92 μM) to 5.36 μM (5.18–5.53 μM), whereas mean (95% CI) effective concentrations for 95% effect (EC_95_) were not significantly different at 7.22 μM (6.09–8.54 μM) and 7.61 μM (7.05–8.20 μM), respectively (*p* = 0.542). When rocuronium-induced block was reversed to a train-of-four (TOF) ratio > 0.9, but with still visible fade, increasing magnesium from 1 mM to 2 mM decreased the TOF ratio to below 0.9. If there was no visible fade after reversal, increasing magnesium concentration did not reduce the TOF ratio.

**Conclusions:**

Magnesium potentiates the neuromuscular effect of rocuronium and shifts the concentration-response curve to the left. Magnesium decreases the safety margin of reversal of rocuronium-induced neuromuscular block with sugammadex.

## Background

Magnesium is one of the most abundant cations in the body and plays a fundamental role as a co-factor in several enzymatic reactions and physiological processes [1, 2]. Its medicinal uses include treatment of arrhythmias [1], bronchospasm [3], pre-eclampsia and eclampsia [1], tocolysis [1], cerebral vasospasm [4], and lately as an adjunct to pain therapy [5].

Magnesium also has several neuromuscular effects, such as decreased liberation of acetylcholine from the presynaptic membrane in the neuromuscular junction [6, 7], a decreased depolarizing effect of acetylcholine on the motor end plate [7] and reduced excitability of the muscle fiber [7]. These effects also influence the action of neuromuscular blocking agents (NMBA): depolarizing NMBAs are antagonized by magnesium [8], while non-depolarizing NMBAs are potentiated, resulting in a faster onset time [9–12] and prolonged clinical effect [9, 12–15]. Several clinical studies found that magnesium administration prior to the injection of NMBA does not significantly impact the efficacy of sugammadex [16, 17], whereas case studies have reported that if magnesium was administered after spontaneous recovery [18] or reversal with sugammadex [19], the significant return of neuromuscular block was seen. A recent pre-clinical study found that while time to recovery was not systematically increased by magnesium for reversal of rocuronium-induced block with equimolar sugammadex, maximal achieved TOF ratio was lower with higher magnesium concentrations [20].

In this study, we used rat phrenic nerve – hemidiaphragm preparations as a controlled ex vivo experimental system to determine the changes in pharmacodynamics caused by magnesium in the rocuronium-sugammadex interaction. The experimental set up allowed us to investigate effects on the neuromuscular junction under near-physiological conditions with constant ion concentrations, while at the same time eliminating such confounding factors as systemic redistribution, drug metabolism and excretion. We hypothesized that unbound rocuronium is potentiated by magnesium, resulting in a reduced effect of sugammadex for reversal of neuromuscular block. Our results highlight the negative impact of magnesium on the safety margin of reversal of neuromuscular block.

## Methods

### Animals, ethics

A total of 20 male Wistar rats from Toxi-Coop Toxicological Research Center, Dunakeszi, Hungary, ranging in weight from 250 to 563 g were used. Institutional guidelines for animal care and usage for research principles were strictly followed. All procedures involving animals were approved by the University of Debrecen Committee of Animal Research (1/2013/DE MÁB). Animals were chosen randomly on the morning of the experiment and euthanized prior to harvesting of tissue specimens.

### Materials

Rocuronium (Esmeron; MSD Pharma Hungary, Budapest, Hungary) and sugammadex (Bridion; MSD Pharma Hungary, Budapest, Hungary) were purchased from commercial vendors and diluted in Krebs-buffer as needed to achieve a dosing volume of 10–100 μl.

Magnesium heptahydrate sulfate (Cormagnesin, Wörwag Pharma GmbH, Böblingen, Germany) was purchased from the commercial vendor and given undiluted to the buffer solution to achieve the indicated final concentrations for magnesium.

### Experimental procedures

The rat phrenic nerve – hemidiaphragm system was used for our experiments. Originally described by Bülbring [21], this technique has been a useful tool to investigate neuromuscular blocking and reversal agents [20, 22, 23]. Rats were given an intraperitoneal overdose of sodium thiopental (60 mg/kg) and exsanguinated through the incision of the dorsal vena cava. Hemidiaphragm preparation was performed by bilateral thoracotomy and removal of the sternum, after which both phrenic nerves were dissected from cranial to rostral direction to the junction with the diaphragm. Then both hemidiaphragms were excised with the corresponding phrenic nerve intact. The hemidiaphragms were then secured in a special tissue holder (ISO-07-TSZ2D, Experimetria Ltd., Hungary) in 75 mL of Krebs-puffer (110 mM NaCl, 5 mM KCl, 1.25 mM CaCl2, 1 mM MgSO4, 1 mM KH2PO4, 5 mM glucose, 20 mM NaHCO3) and aerated by bubbling 95% O2 + 5% CO2 (Vol%) through the solution. The solution was maintained at a temperature of 37 °C (AMP-09 Temperature controller, Experimetria Ltd., Hungary).

The hemidiaphragms were attached to an isometric force-displacement transducer (FSG-01/200 Force Transducer, Experimetria Ltd.) at the centrum tendineum using a commercially available 5/0 diameter surgical thread. Measurements were amplified by AMP-01-SG Classic bridge amplifier and recorded with the S.P.E.L. Advanced Isosys software (Experimetria Ltd., Hungary). The phrenic nerve was stimulated either with a single twitch every 5 s (rectangular pulses with 0.3 ms pulsewidth and supramaximal voltage) or a 2-Hz train-of-four (TOF) stimulus every 15 s (rectangular pulses of 0.2 ms duration with a supramaximal voltage) using a square wave stimulator (ST-03-O4, Experimetria Ltd., Hungary).

**Fig. 1:**
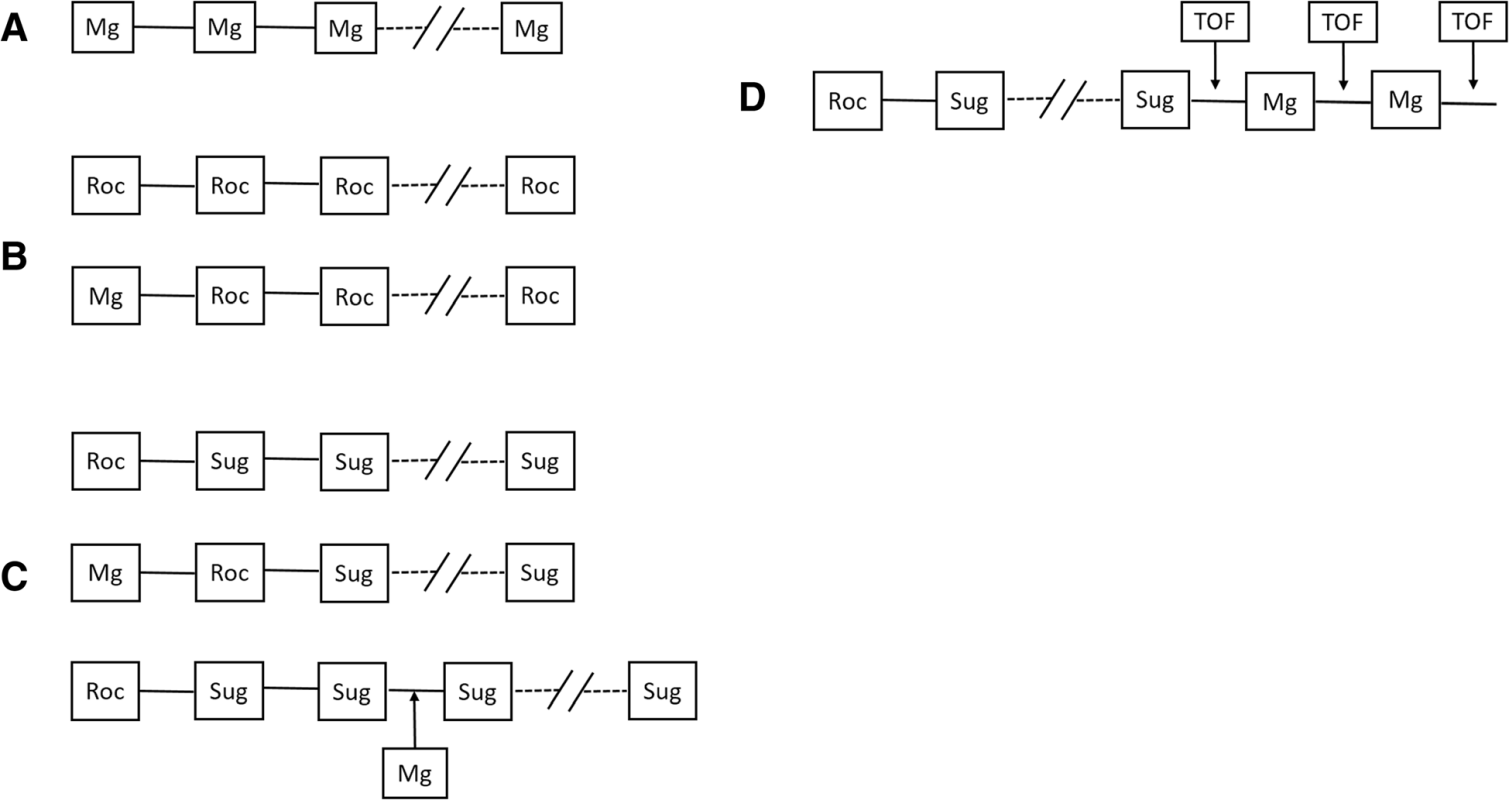
Study design. **a** Concentration-response curve for magnesium. Magnesium doses were added every 10 min to the buffer solution until the suppression of single twitch force amplitude was achieved. **b** Concentration-response curve for rocuronium. Rocuronium boluses were added every 15 min until the suppression of single twitch force amplitude was achieved. To assess the effect of magnesium, the experiment was repeated with a magnesium bolus given 10 min prior to commencing rocuronium dosing. **c** Concentration-response curve for sugammadex. After an initial bolus of rocuronium which caused at least 90% suppression of single twitch force amplitude, sugammadex doses were given every 10 min until full recovery of single twitch force amplitude was achieved. To assess the effect of magnesium given before neuromuscular block, the experiment was repeated with a magnesium bolus given 10 min prior to administering the rocuronium dose. To assess the effect of magnesium given after the establishment of neuromuscular block, the experiment was repeated with a magnesium bolus given after partial reversal with sugammadex and then sugammadex dosing was continued. **d** Effect of magnesium on the safety margin of reversal. An initial rocuronium dose was followed by incremental doses of sugammadex to achieve either a “full” or clinically “accepted” reversal, as measured by the train-of-four (TOF) ratio. Magnesium was given in increments and the TOF ratio reassessed after every change in magnesium concentration. Mg: magnesium, Roc: rocuronium, Sug: sugammadex, TOF: measurement of train-of-four ratio

After submersion in buffer solution, the tissue preparations were allowed an acclimatization period of 10 min without stimulation at an applied tension of 20–30 mN. Then stimulation was started and an additional 1–1.5 h without treatment (with buffer changes as needed) followed until a stable baseline tension was achieved. Drug dosing was only commenced after this stabilization period (for a scheme of drug dosing and study design, see Fig. 1). After the measurement of a given concentration-response curve, the buffer solution was removed 5 times in a 30-min timespan to assure complete washout of any agents before measuring a new curve.

Each concentration-response relationship shown in the figures is based on 5 concentration-response curves, each from a different specimen. Whereas multiple concentration-response curves were measured on a given specimen, a specific concentration-response curve was measured only once, thus each specimen only contributed one data set to a given concentration-response relationship. To mitigate the effects of degradation of the tissue specimen over time, the order of concentration-response curve experiments was permuted between specimens. The specimen was discarded if a stable baseline tension was no longer attainable.

#### Concentration-response curves for magnesium and rocuronium

The effects of magnesium and rocuronium were quantified as the depression of single twitch force amplitude. The twitch force amplitude for a measurement point was the mean value of five consecutive contractions at a given drug concentration once contraction amplitude had stabilized corrected with the baseline tension. The twitch force amplitude was normalized to the maximum twitch force amplitude of the untreated sample to construct cumulative concentration-response curves.

In the case of magnesium, 9.2 mg was administered every 10 min to the buffer solution until full depression of the twitch amplitude. Each specimen contributed 11–12 measurement points to the curve.

For rocuronium, a dose was given every 15 min. We constructed two curves, one for [Mg^2+^] = 1 mM and the other for [Mg^2+^] = 1.5 mM. For the case of [Mg^2+^] = 1 mM the initial dose of rocuronium was 0.2 mg followed by doses of 0.1 mg until contraction was no longer detectable after nerve stimulation. Each specimen contributed 5–7 measurement points to the curve. For the case of [Mg^2+^] = 1.5 mM, the first dose was 0.1 mg, then two doses of 0.05 mg followed by doses of 0.025 mg until contraction was no longer detectable after nerve stimulation. Each specimen contributed 7–11 measurement points to the curve.

#### Pre-block effect of magnesium

To determine the pre-block effect of magnesium on reversal of neuromuscular block, a single rocuronium dose (0.3 mg rocuronium for [Mg^2+^] = 1.5 mM and 0.5 mg rocuronium for [Mg^2+^] = 1 mM) was given to achieve a 90–95% depression of single twitch force amplitude. Then sugammadex doses were given in 10-min intervals. Twitch force amplitude was baseline corrected with the twitch force amplitude of the sample prior to reversal with sugammadex and normalized to the maximum contraction amplitude after full reversal to construct cumulative concentration-response curves. For [Mg^2+^] = 1 mM an initial sugammadex dose of 0.2 mg was given, followed by four doses of 0.1 mg, then 0.15 mg, which was followed by doses of 0.25 mg until twitch amplitude plateaued and reversal of neuromuscular block was verified by achieving a TOF ratio (T4/T1 of the four stimuli) > 0.9. Each specimen contributed 5–8 measurement points to the curve. For [Mg^2+^] = 1.5 mM an initial sugammadex dose of 0.05 mg was given, followed by three doses of 0.1 mg, then 0.15 mg, which was followed by doses of 0.25 mg until twitch amplitude plateaued and reversal of neuromuscular block was verified by achieving a TOF ratio (T4/T1 of the four stimuli) > 0.9. Each specimen contributed 7 measurement points to the curve.

#### Post-block effect of magnesium

To determine the post-block effect of magnesium, rocuronium 0.5 mg was given at [Mg^2+^] = 1 mM to achieve a 90–95% depression of single twitch force amplitude. Reversal was started with incremental doses of 0.1–0.2 mg sugammadex every 10 min until a cumulative dose of 0.5 mg was achieved. At this point [Mg^2+^] was increased to 1.5 mM and sugammadex dosing was continued with one dose of 0.15 mg, followed by one dose of 0.1 mg. This was followed by doses of 0.25 mg until twitch amplitude plateaued and reversal of neuromuscular block was verified by achieving a TOF ratio > 0.9. Twitch force amplitude was baseline corrected with the twitch force amplitude of the sample prior to reversal with sugammadex and normalized to the maximum contraction amplitude after full reversal to construct cumulative concentration-response curves. Each specimen contributed 6–7 measurement points to the curve.

#### Effect of magnesium on the safety margin of reversal

The effect of magnesium on the safety margin of reversal was investigated by reversing rocuronium-induced neuromuscular block with sugammadex until either the TOF ratio was ≈ 1.0 without visible fade (“full” reversal) or TOF ratio was > 0.9, but still showed a visible fade (“accepted” reversal [24]). Then [Mg^2+^] was increased in 0.5 mM steps from 1 mM to 2 mM. TOF ratio was recorded every 15 s for 10 min after a change in [Mg^2+^] and the final stable TOF ratio was used to quantify the change in the TOF ratio.

### Statistical analysis

GraphPad Prism 6 for Windows (GraphPad Software, Inc., La Jolla, CA, USA) was used for fitting of concentration-response curves. Curve fitting was done by nonlinear regression with either the “log (agonist) vs. normalized response – variable slope” or the “log (inhibitor) vs. normalized response – variable slope” function. The fitting equation was: y = 100/(1 + 10^((LogEC50-X)*HillSlope)), where X is the log_10_ value of concentration and y is the normalized and baseline corrected contraction amplitude.

For sample size determination, we performed a pilot study (*n* = 3) to determine the concentration-response curve of rocuronium. We arrived at values of logEC50 = 0.875 and SD = 0.023. Assuming a 10% change in EC50 as clinically relevant, group sample size at α = 0.05 and power of 80% was 4 for a one-sided test. Statistical comparison of concentration-response curves was done with GraphPad Prism 6 for Windows with the extra sum-of-squares F-test. LogEC50 was the model component that was used to account for the extra sum-of-squares. Results are presented as mean and 95% confidence interval unless otherwise specified.

## Results

### The effect of magnesium on the diaphragm contraction force

**Fig. 2:**
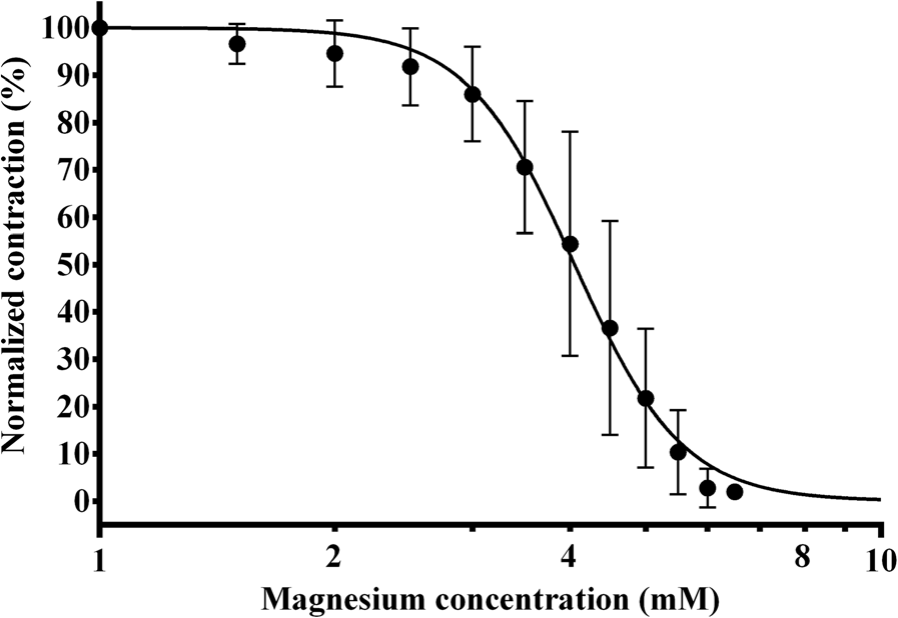
The concentration-response curve of magnesium. Normalized contraction force amplitude as a function of magnesium concentration on a logarithmic scale (logarithm to base 10/ log_10_). The curve is the best fit curve calculated from measurements on *n* = 5 different specimens by non-linear regression. Measurement points represent mean values of normalized contraction force amplitude at a given concentration. Error bars show the standard deviation of measurement points

As a first step, we determined the concentration-response curve of magnesium alone (Fig. 2). The effective concentration for 50% effect (EC_50_) was 4.06 mM (95% CI: 3.91–4.21 mM). From the concentration-response curve, we can see that increasing the concentration of magnesium from 1 mM to 1.5 mM resulted in a mean reduction of contraction force amplitude to 96.6% (95% CI: 91.4–101.8%) of the amplitude measured with 1 mM magnesium concentration.

### The effect of magnesium on rocuronium-induced neuromuscular block

**Fig. 3:**
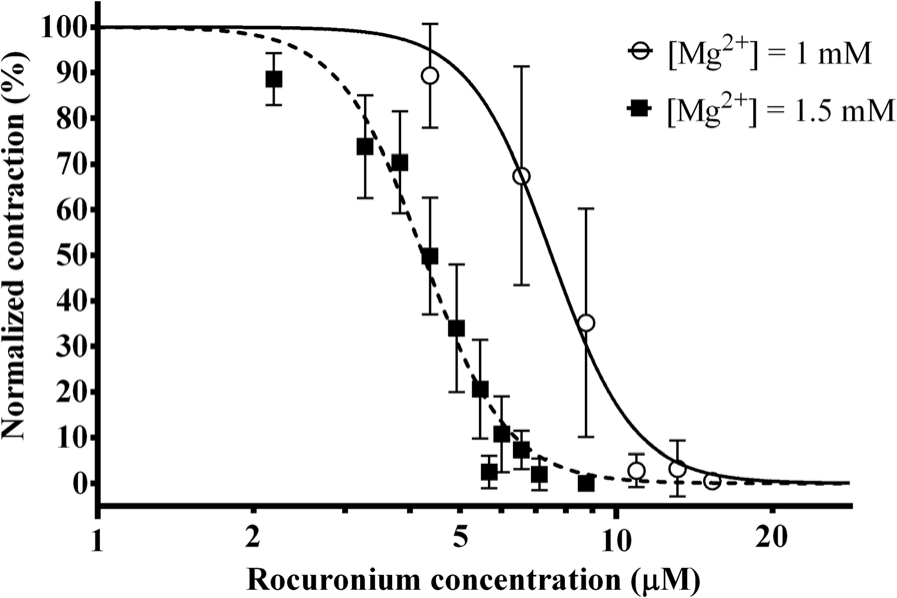
The concentration-response curve of rocuronium at magnesium concentrations of 1 and 1.5 mM. Normalized contraction force amplitude as a function of rocuronium concentration on a logarithmic scale (logarithm to base 10). The curve is the best fit curve calculated from measurements on *n* = 5 different specimens by non-linear regression. Measurement points represent mean values of normalized contraction force amplitude at a given concentration. Error bars show the standard deviation of measurement points

The concentration-response curve of rocuronium was determined with a magnesium concentration of either 1 or 1.5 mM in the buffer solution (Fig. 3). Increasing the magnesium concentration resulted in a left-shift of the concentration-response curve. The EC_50_ values with a magnesium concentration of 1 or 1.5 mM were 7.50 μM (6.93–8.12 μM) and 4.26 μM (4.09–4.43 μM), respectively (*p* < 0.0001). The effective concentrations for 95% effect (EC_95_) were 12.89 μM (10.67–15.56 μM) and 7.35 μM (6.64–8.13 μM), respectively (*p* < 0.0001). Best fit values of concentration-response curves are summarized in Table 1.

**Table 1:** Best fit values of concentration-response curves for rocuronium and sugammadex

Curve	Mean (95% CI) EC_50_	Mean (95% CI) EC_95_
Magnesium	4.06 (3.91–4.21) mM	
Rocuronium ([Mg^2+^] = 1 mM)	7.50 (6.93–8.12) μM	12.89 (10.67–15.56) μM
Rocuronium ([Mg^2+^] = 1.5 mM)	4.26 (4.09–4.43) μM	7.35 (6.64–8.13) μM
	*p* < 0.0001	*p* < 0.0001
Sugammadex (Rocuronium 0.5 mg, [Mg^2+^] = 1 mM)	3.67 (3.43–3.92) μM	7.22 (6.09–8.54) μM
Sugammadex (Rocuronium 0.3 mg, [Mg^2+^] = 1.5 mM)	1.51 (1.42–1.60) μM	4.48 (3.80–5.29) μM
	*p* < 0.0001	*p* < 0.002
Sugammadex (Rocuronium 0.5 mg, [Mg^2+^] = 1 + 0.5 mM)	5.36 (5.18–5.53) μM	7.61 (7.05–8.20) μM
	*p* < 0.0001	*p* = 0.542

The displayed *p*-values are in comparison with the reference concentration-response curve measured with [Mg^2+^] = 1 mM. EC_50_: effective concentration for 50% effect, EC_95_: effective concentration for 95% effect, CI: confidence interval

### The effect of magnesium on the reversal of rocuronium-induced neuromuscular block with sugammadex

**Fig. 4:**
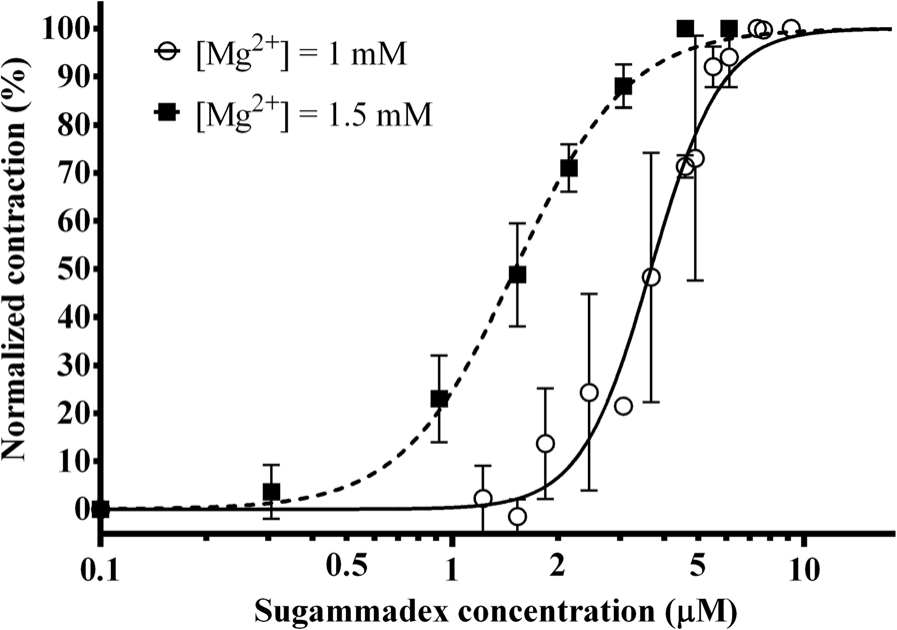
The concentration-response curve of sugammadex for reversing rocuronium-induced block at pre-block magnesium concentrations of 1 and 1.5 mM. Normalized contraction force amplitude as a function of sugammadex concentration on a logarithmic scale (logarithm to base 10). Due to potentiation of neuromuscular block by magnesium, dose of rocuronium for achieving block was either 0.3 mg ([Mg2+] = 1.5 mM) or 0.5 mg ([Mg2+] = 1 mM). The curve is the best fit curve calculated from measurements on *n* = 5 different specimens by non-linear regression. Measurement points represent mean values of normalized contraction force amplitude at a given concentration. Error bars show the standard deviation of measurement points

Neuromuscular block was induced with rocuronium with a magnesium concentration of either 1 or 1.5 mM in the buffer solution and then reversed with incremental doses of sugammadex (Fig. 4). Due to potentiation of neuromuscular block, rocuronium dose had to be modified with the higher magnesium concentration to achieve the same level of block as with the lower magnesium concentration. Therefore rocuronium dose was reduced from 0.5 mg with a magnesium concentration of 1 mM to 0.3 mg for a magnesium concentration of 1.5 mM. The EC_50_ values of sugammadex with a magnesium concentration of 1 or 1.5 mM were 3.67 μM (3.43–3.92 μM) and 1.51 μM (1.42–1.60 μM), respectively. The EC_95_ values were 7.22 μM (6.09–8.54 μM) and 4.48 μM (3.80–5.29 μM), respectively. The differences in EC_50_ and EC_95_ were statistically significant (*p* < 0.0001 for EC_50_ and *p* = 0.002 for EC_95_).

**Fig. 5:**
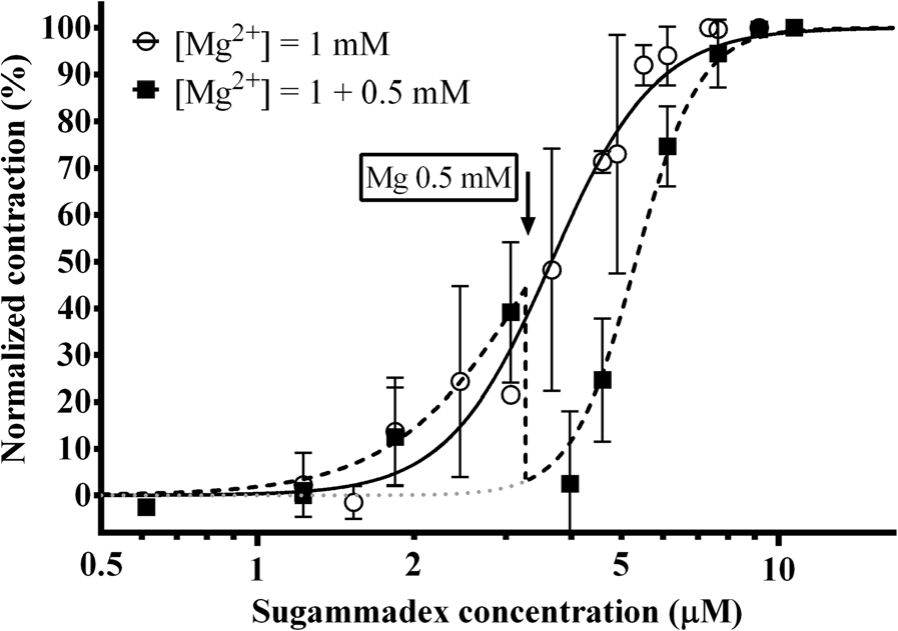
Effect of post-block administration of magnesium on the concentration-response curve of sugammadex for reversing rocuronium-induced block. Normalized contraction force amplitude as a function of sugammadex concentration on a logarithmic scale (logarithm to base 10). After an initial dose of 0.5 mg rocuronium at [Mg2+] = 1 mM, the block was reversed by sugammadex at either unchanged magnesium concentration (○) or by increasing [Mg2+] to 1.5 mM after partial reversal (■). Magnesium concentration was only increased after partial reversal (indicated by black arrow) to be able to visualize contractions despite potentiation of the block by magnesium. The black dashed line (⁃⁃⁃**)** shows actual normalized contraction force amplitude, whereas the grey dotted line (● ● ●) is the best fit curve calculated from measurements on *n* = 5 different specimens by non-linear regression. Measurement points represent mean values of normalized contraction force amplitude at a given concentration. Error bars show the standard deviation of measurement points

To assess the effect of post-block administration of magnesium, magnesium was increased after partial reversal of rocuronium-induced block with sugammadex (Fig. 5). Increasing magnesium concentration in this setting resulted in a sharp drop in contraction force amplitude and a corresponding increase in EC_50_ of sugammadex from 3.67 μM (3.43–3.92 μM) to 5.36 μM (5.18–5.53 μM), which was a statistically significant difference (*p* < 0.0001). However, EC_95_ values were statistically non-significantly different (*p* = 0.542) at 7.22 μM (6.09–8.54 μM) and 7.61 μM (7.05–8.20 μM).

### Effect of magnesium on the safety margin after reversal of neuromuscular block with sugammadex

**Fig. 6:**
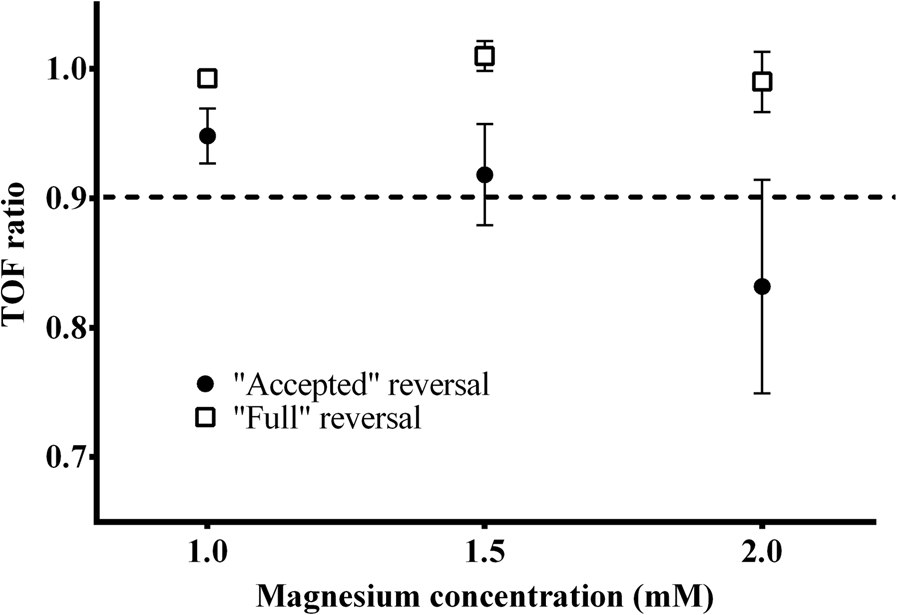
Magnesium-induced change in the train-of-four (TOF) ratio. Train-of-four ratio as a function of magnesium concentration. After an initial dose of rocuronium 0.5 mg, sugammadex was given in incremental doses to either achieve a clinically “accepted” (1 > TOF > 0.9, but with still visible fade) or “full” reversal (TOF ≈ 1.0, no visible fade). Magnesium concentration was then increased to 1.5 mM and then again to 2 mM. For each reversal type *n* = 5 different specimens were measured. Measurement points represent mean values of TOF ratio at a given concentration. Error bars show the standard deviation of measurement points

Since the addition of magnesium after partial reversal with sugammadex resulted in a decrease in contraction force amplitude and an increase in the steepness of the concentration-response curve, we wanted to investigate whether traditional measures of safe reversal of neuromuscular block are still reliable under the condition of changing magnesium concentrations. Therefore neuromuscular block was reversed to achieve either “full” reversal (mean TOF ratio: 0.99 ± 0.006, no visible fade) or a clinically “accepted” safe reversal (TOF ratio > 0.9; mean TOF ratio: 0.95 ± 0.02, but with visible fade) and then magnesium concentration was increased incrementally (Fig. 6). The sugammadex to rocuronium concentration ratio was 1.43 (0.83–2.02) in the “full” reversal group and 0.68 (0.60–0.76) in the clinically “accepted” reversal group. Whereas TOF ratio remained stable with increases in magnesium concentration in the “full” reversal group, we witnessed a gradual decline in TOF ratio and the return of neuromuscular block to below a TOF ratio of 0.9 in the clinically “accepted” reversal group.

## Discussion

In this ex vivo study, we investigated the neuromuscular effects of magnesium and its interaction with rocuronium and sugammadex. In agreement with previous studies, magnesium in itself induced dose-dependent suppression of muscle contraction force [20, 25]. The mechanism of magnesium’s neuromuscular effect is multifactorial, including decreased liberation of acetylcholine from the presynaptic membrane in the neuromuscular junction [7] and a decreased depolarizing effect of acetylcholine on the motor end plate [7]. These effects can be reversed by increasing the calcium concentration, implicating antagonism of calcium as an underlying cause [7].

Magnesium also caused a left-shift in the concentration-response curve of rocuronium, similar to its effect on d-turbocurarine [25]. The interaction between magnesium and rocuronium appears to be synergistic, as the reduction in muscle contraction force exceeds the sum of isolated rocuronium and magnesium effect. Previously it was suggested that this might be attributed to decreased liberation of acetylcholine from presynaptic nerve terminals due to an inhibition a voltage-gated calcium channels by magnesium [20], however, there is also in vitro evidence for receptor-level synergism in the case of vecuronium and magnesium [26].

Upon first inspection, pre-block administration of magnesium enhanced the actions of sugammadex as well. However, one has to consider the dose-sparing effect of magnesium, resulting in lower rocuronium concentrations to achieve the same level of neuromuscular block. Therefore the amount of NMBA sugammadex has to bind is less, which might explain the apparent left-shift in the concentration-response curve of sugammadex. Conversely, if we increased the concentration of magnesium only after commencing reversal of block (at partial recovery), the potentiation of rocuronium-induced neuromuscular block by additional magnesium increased the depth of block and shifted the concentration-response curve of sugammadex to the right. This effect might be attributed to the synergism between magnesium and unbound rocuronium molecules. However, even under these circumstances, we saw no significant increase in the full reversal dose of sugammadex. Although we cannot entirely rule out an interaction between magnesium and sugammadex from our data, previous microcalorimetry experiments failed to show any affinity between magnesium and sugammadex [27]. Therefore, the likelihood of a clinically significant interaction between magnesium and sugammadex is small and probably not relevant when compared with the more pronounced potentiation of rocuronium’s effect by magnesium.

A previous case report with vecuronium found that magnesium sulphate administration after spontaneous recovery to a TOF ratio > 0.7 was able to induce a profound return of neuromuscular block [18]. The same phenomenon was described for a patient who received a large dose of magnesium for the treatment of atrial fibrillation after rocuronium-block was reversed with sugammadex [19]. Our own results show that even with a reversal to an accepted safe level [17] (TOF ratio > 0.9) this effect persists and only complete reversal prevents the magnesium-induced return of neuromuscular block. The degree of the recurrent block can be sufficient to revert a previously safely reversed neuromuscular block to the level of unsafe to extubate (TOF ratio < 0.9). The change in the concentration of magnesium necessary for this degree of TOF depression can be achieved with relatively small doses, as even the highest concentration in our study was still in the near-physiological range. The sugammadex to rocuronium concentration ratio shows that with full reversal there is a surplus of sugammadex molecules, so given the 1:1 binding ratio of sugammadex for rocuronium [27], all rocuronium molecules are encapsulated. However, if the neuromuscular block is only reversed to TOF ratio > 0.9, a significant fraction of rocuronium molecules will not be encapsulated by sugammadex. Our results indicate 25–40% of rocuronium molecules are not bound in this scenario, which is in line with results from a mouse system [27]. Likely the potentiation of these free rocuronium molecules results in the decrease in TOF ratio following magnesium administration.

The ex vivo nature of our study poses limitations with regards to the direct clinical applicability of the results. One major confounder is that through a large portion of the preparation process the tissue specimen is in a hypoxic state. At present we do not know what kind of receptor level changes this might induce or how this might affect intracellular ion trafficking and utilization. However, any such effects should be uniform among specimens as they were harvested in a similar fashion. The distinct advantage of this ex vivo system is that it allows the precise quantitative characterization of phenomena to a degree that is hard to achieve in in vivo, let alone clinical studies. Connections and interactions that are otherwise masked by the gross dosing of study drugs (relative to the concentration-response curve) or study endpoints that are only surrogates of actual efficacy (i.e. time to reversal of block with a fixed dose of sugammadex as opposed to an actual concentration-response relationship) can be thus uncovered. If the available clinical data are interpreted in the context of our ex vivo findings, several clinically relevant assumptions regarding the effects of magnesium can be made.

When magnesium is given prior to initiation of neuromuscular block and there is no compensatory reduction in non-depolarizing NMBA dose, then potentiation and left-shift of the concentration-response curve will cause a faster onset time and a prolonged block, as has already been shown for vecuronium and rocuronium [9, 11, 12]. While one could expect potentiation of neuromuscular block by magnesium pretreatment to prolong reversal with sugammadex, two clinical studies failed to show any significant effect [16, 17]. An ex vivo study also came to the conclusion that increasing magnesium concentration does not systematically increase reversal time by sugammadex [20].

When magnesium is administered after the non-depolarizing NMBA or during a/following reversal, then total reversal dose of sugammadex is still unchanged, however, the degree of neuromuscular block caused by unbound NMBAs will be increased [28]. Magnesium given after incomplete spontaneous recovery or pharmacological reversal can cause a recurrent neuromuscular block, even if the TOF ratio is > 0.9 [19, 29]. The degree of the block can be clinically significant even if the concentration of magnesium remains in the near-physiological range. Therefore magnesium should be administered carefully postoperatively and quantitative neuromuscular monitoring is advised to confirm full reversal and exclude return of neuromuscular block, even when using sugammadex.

## Conclusion

In our ex vivo system magnesium exhibited a marked enhancement of the neuromuscular block caused by rocuronium and shifted the concentration-response curve of rocuronium to the left. Although the interaction of sugammadex and rocuronium was not significantly influenced by magnesium, the potentiation of unbound rocuronium by magnesium caused a decrease in the safety margin of reversal and was only prevented by a full reversal of neuromuscular block by sugammadex.

## Abbreviations

NMBA: Neuromuscular blocking agents
TOF: Train of four
